# Vaccinating Meat Chickens against *Campylobacter* and *Salmonella:* A Systematic Review and Meta-Analysis

**DOI:** 10.3390/vaccines10111936

**Published:** 2022-11-15

**Authors:** Adriana C. Castelo Taboada, Anthony Pavic

**Affiliations:** Birling Laboratories Pty. Ltd., 975 The Northern Road, Bringelly, NSW 2556, Australia

**Keywords:** bacteria, caeca, immunisation, poultry, broiler, enteropathogen

## Abstract

Foodborne enteritis is a major disease burden globally. Two of the most common causative bacterial enteropathogens in humans are *Campylobacter* and *Salmonella* species which are strongly associated with the consumption of raw or contaminated chicken. The poultry industry has approached this issue by use of a multi-hurdle method across the production chain to reduce or eliminate this risk. The use of poultry vaccines is one of these control methods. A systematic review and meta-analysis of vaccination effects against caecal *Campylobacter* and *Salmonella* were performed on primary research published between 2009 and 2022. Screening was conducted by three reviewers with one reviewer performing subsequent data extraction and one reviewer performing the risk of bias assessment. The confidence in cumulative evidence was evaluated based on the GRADE method. Meta-analyses were performed using standardised mean differences (SMDs) with additional analyses and random effects regression models on intervention effects grouped by the vaccine type. A total of 13 *Campylobacter* and 19 *Salmonella* studies satisfied the eligibility criteria for this review. Many studies included multi-arm interventions, resulting in a total of 25 *Campylobacter* and 34 *Salmonella* comparators which were synthesised. The analyses revealed a large reduction in pathogen levels; however, many effects required statistical adjustment due to unit of analysis errors. There was a moderate level of confidence in the reduction of *Campylobacter* by 0.93 SMD units (95% CI: −1.275 to −0.585; *p* value < 0.001) and a very low level of confidence in the reduction of *Salmonella* by 1.10 SMD units (95% CI: −1.419 to −0.776; *p* value < 0.001). The Chi^2^ test for heterogeneity (*p* value 0.001 and <0.001 for *Campylobacter* and *Salmonella,* respectively) and the I^2^ statistic (52.4% and 77.5% for *Campylobacter* and *Salmonella*, respectively) indicated high levels of heterogeneity in the SMDs across the comparators. The certainty of gathered evidence was also affected by a high risk of study bias mostly due to a lack of detailed reporting and, additionally for *Salmonella*, the presence of publication bias. Further research is recommended to source areas of heterogeneity, and a conscious effort to follow reporting guidelines and consider units of analysis can improve the strength of evidence gathered to provide recommendations to the industry.

## 1. Introduction

Foodborne enteritis is a major disease burden globally and can be caused by a variety of pathogens and pollutants [1]. Of the bacterial infectors, two of the most common human enteropathogens are *Campylobacter* and *Salmonella* with case rates (per 100,000 people) of: USA 14.4 and 13.3 [2], EU 64.8 and 19.7 [3] and Australia 146.9 and 74.7 [4], respectively. *Campylobacter* and *Salmonella* food poisoning are mostly associated with the consumption or cross-contamination of chicken meat products [5,6].

*Campylobacter* is Gram-negative, micro-aerophilic, motile, curved rod bacteria and comprises several species with *jejuni* and *coli* being zoonotic causes of gastroenteritis in humans [7]. The chicken gut, especially the caeca, is the ideal growth environment for high levels of *Campylobacter*; however, *Campylobacter* does not remain viable outside its specific growth conditions, making it difficult to culture from the environment and to implement control strategies [8,9]. Horizontal transmission of *Campylobacter* occurs so quickly within the Australian meat chicken industry that it is uncommon for a flock to be negative for *Campylobacter* once it reaches the point of slaughter [10].

*Salmonella* is Gram-negative, facultative anaerobic, predominantly motile, rod bacteria of two species, *bongori* and *enterica,* with the majority of serovars found in the *enterica* species [11]. *Salmonella* serovars predominantly colonise the chicken gut in low levels but can also survive as viable bacteria in the environment [12]. *Salmonella* transmission in chickens can occur horizontally or vertically; however, colonisation in meat chickens is not stable with a general flock prevalence of 10–30% [13].

There are many points within the poultry food chain where control measures for *Campylobacter* and *Salmonella* can be implemented. The Codex: Guidelines for the control of *Campylobacter* and *Salmonella* in Chicken Meat CAC/GL 78-2011 [14,15] and FSIS guidelines for controlling *Salmonella* and *Campylobacter* in raw poultry [16,17] provide practical interventions to control contamination within several stages across the poultry food chain.

The use of vaccines during meat chicken primary production is an adjunct method used to reduce pathogen populations and subsequent risk to the consumer [18]. This intervention stimulates the chicken’s immune system to produce antibodies to protect them from enteropathogenic colonisation and was reviewed by Pumtang-On in 2021 [19] and Totton et al., in 2012 [20], for *Campylobacter* and *Salmonella*, respectively. Pumtang On and colleagues [19] found study experimental design and *Campylobacter* vaccine effects were very inconsistent and did not synthesise the results in a meta-analysis. Totton et al. [20] found beneficial effects of *Salmonella* live vaccines; however, the strength of evidence was limited due to the low number of eligible studies found in the literature. Although the use of *Salmonella* vaccines is common practice, there are no commercially available *Campylobacter* vaccines for poultry.

Research into the optimisation, development and introduction of safe and effective *Campylobacter* and *Salmonella* vaccines is rapidly growing globally, and there is a need to synthesise all available evidence to thoroughly understand or determine how effective this is as a control measure for poultry risk assessors. The objective of this systematic review is to investigate the effectiveness of vaccinating meat chickens on caecal *Campylobacter* or *Salmonella* and statistically synthesise the effects in a meta-analysis.

## 2. Materials and Methods

### 2.1. Scope

This study is an updated sub-component of a larger project that explored the effects of interventions delivered at the primary production, processing or distribution channel stages in reducing *Campylobacter* and *Salmonella* levels in meat chickens. This review was performed on studies that used vaccines to address *Campylobacter* and *Salmonella* colonisation in meat chickens.

### 2.2. Eligibility Criteria

Studies that were included in the broad review were limited to meat chickens (live or in ovo) or products as well as surfaces exposed to meat chicken/product handling. Eligible studies applied controlled interventions at any stage during meat chicken production or processing prior to mincing, marination or any other value-adding step and measured outcomes as *Campylobacter* and *Salmonella* levels or prevalence in detail. The studies had a randomised trial design that described intervention methods in sufficient detail for implementation. Studies were primary research, published within 2009 to 2019 in English (due to insufficient budget for translation), and were not limited by region.

Data extracted from the broad review were used to further screen for studies that used vaccines or immunisation methods on meat chickens and measured endpoint caecal *Campylobacter* and *Salmonella* Additional screening was also performed on studies published within 2019 to 2022 to form this updated review.

### 2.3. Information Sources

The initial database search was performed on 29 October 2019 where “Interface/URL (Date) (Database/Information Source)”: “Web of Science (29 October 2019) (Science Citation Index—Expanded Conference Proceedings Citation Index—Science, Medline and CAB Abstracts)”; “USDA National Agricultural Library Citation Database: https://agricola.nal.usda.gov/ (24 December 2019) (Agricola)”; “Informit (29 October 2019) (ANR-Index, ANR-Index Archive)”; “FAO: http://agris.fao.org/agris-search/ (9 January 2020) (Agris [1975–])”; “Proquest (9 January 2020) (Proquest Dissertations and Theses Global)”; “Trove: National Library of Australia (9 January 2020) (Australian Theses)”. The search domain was limited to Title, Abstract and Keyword fields for articles published in the 10 years inclusive of January 2009 to November 2019 to cover practices that were either presently relevant or novel. Search terms (or derivations of) used were: (broiler* OR chicken* OR gallus* OR poultry* OR “meat bird*”) AND (control* OR reduc* OR hygien* OR risk* OR eliminat*) OR “sanitary dressing” OR “slaughter hygiene” OR “hygiene dressing”) AND (salmonell* OR campy* OR enteropath*).

The additional search for literature published within November 2019 to 13 July 2022 was performed on 13 July 2022 using Informit, Web of Science, USDA Agricola and AGRIS. The search domain was limited to the abstract field using search terms (broiler* OR chicken* OR gallus* OR poultry* OR “meat bird*”) AND (vaccin* OR immuni* OR antibod*) AND (salmonell* OR campy*).

### 2.4. Screening Procedures

Two reviewers independently performed relevance screening (title and abstract screening and full-text screening) using Covidence software [21], and duplicate studies were removed at both stages.

Titles and abstracts were screened against the eligibility criteria for the population, intervention, comparator and outcome, prior to progression to full-text screening. Abstracts where both reviewers scored YES, or at least one reviewer scored UNCLEAR, moved to full-text screening. Disagreements were resolved by a third reviewer, and where consensus could not be reached, the abstract advanced to full-text screening.

At the full-text screening stage, studies were screened against all the eligibility criteria and excluded if the full text was unobtainable. Studies progressed to the data extraction/risk of bias assessment stage when both reviewers scored YES. Disagreements were resolved by discussion.

### 2.5. Data Extraction

Data were extracted from the included studies, and results presented in graphs were extracted using the free-to-use software ycasd version 3 as described by Gross and colleagues [22]. Authors were not contacted for missing data or for clarification of published results. Data extracted included: (1) bibliographic information: study name, authors, publication title and year, country, institution and design (i.e., level of allocating units to treatment groups); (2) population characteristics: chicken breed, age and sex; (3) intervention protocols: type, administration method, dose, frequency, duration and control type (i.e., non-treated/placebo/standard treatment); (4) challenge details (if applicable): level, time and type of challenge; (5) outcome details: method of outcome assessment, method of adjustment for non-independence in outcome data (if applicable) and reported point estimates with variability measures.

### 2.6. Risk of Bias

The risk of bias (RoB) in individually randomised studies was assessed using the Risk of bias version 1 tool, implemented in Covidence [23]. An additional domain of bias arising from the timing of identification and recruitment of subjects was assessed using guidance from the Cochrane risk-of-bias tool for cluster-randomised and randomised cross-over trials [24]. Other sources of bias described in SYRCLE’s “Risk of bias” tool for animal studies were accounted for in the “Other bias” domain [25]. One reviewer judged each study’s comparators as being at low, high or unclear risk of bias for the required domains to provide an overall judgement for the comparator as per methods outlined in the GRADE Handbook [26].

### 2.7. Data Synthesis

Characteristics of included studies were summarised, and eligible studies were selected for synthesis in a meta-analysis.

The primary meta-analyses were performed to answer the following questions: Do vaccines or immunisations against *Campylobacter* reduce *Campylobacter* in the caeca of meat chickens compared to no vaccination or immunisation? Do vaccines or immunisations against *Salmonella* reduce *Salmonella* in the caeca of meat chickens compared to no vaccination or immunisation? Additional subgroup analyses were performed to answer the following: Do the specific intervention types modify the magnitude of the intervention effects?

To combine the results across all studies, Hedge’s (adjusted) G standardised mean difference (SMD) was chosen as the effect measure to account for the small sample bias common in animal studies. Summary statistics were transformed into the appropriate input values required for Stata’s metan command [27,28]. Odds ratios of prevalence data were transformed to SMDs, and a correction of 0.5 was used when studies recorded no events. Data extracted from studies that used multiple similar (e.g., different doses of the same treatment) interventions were pooled [29]. If different treatments were used, the sample size of the shared control group was split to avoid “double-counting” [29]. SMDs from multiple time points on the same population were pooled by averaging the SMDs and variances of the SMDs [30].

Standardised mean differences were meta-analysed using the metan package in Stata 13 [27,28]. A random effects model using the DerSimonian and Laird variance estimator [31] and Wald-type confidence intervals was chosen given the anticipated diversity in the study characteristics. The 95% prediction intervals were used to predict the true effects of the treatments in similar individual studies.

Statistical heterogeneity was assessed by visually inspecting overlap of effect estimate confidence intervals across comparators in the forest plot, then by a Chi^2^ test for heterogeneity. Inconsistency was evaluated using the I^2^ statistic with criteria described in Chapter 10 of the Cochrane Handbook for Systematic Reviews [32]. If the I^2^ statistic fell in an area where the criteria overlap, the magnitude, direction and heterogeneity of the effects were considered to evaluate the importance of the inconsistency. Funnel plots were used to assess reporting bias of the studies with contour-enhanced funnel plots to inspect where effects lay among areas of statistical (non)significance. Random effects subgroup analyses with meta-regression model (Bonferroni adjusted) *p* values were used to investigate interactions between the intervention and subgroups.

A sensitivity analysis was performed using adjusted SMDs calculated from effective sample sizes for studies that did not adjust for within-cluster correlation. The originally extracted sample sizes were replaced by effective sample sizes estimated using an intra-cluster correlation (ICC). Due to a lack of available ICCs for vaccinations for *Campylobacter* or *Salmonella* in meat chicken ceca, an ICC of 0.1 was used to account for smaller clusters typical in animal trials [33]. Studies with missing data (e.g., reporting of summary statistics that could not be transformed into an SMD or OR) were included in the review but excluded from the meta-analysis.

The certainty in findings was assessed using the Grading of Recommendations, Assessment, Development and Evaluations (GRADE) approach [26]. This method used five domains (Risk of Bias, Imprecision, Inconsistency, Indirectness and Publication Bias) to produce an overall certainty rating of: very low, low, moderate and high. Assessment began at high certainty as studies were randomised controlled trials. The certainty of evidence was rated down accordingly by one or two levels if the potential limitations lowered the confidence in the estimated effect.

## 3. Results

### 3.1. Search Result

**Figure 1:**
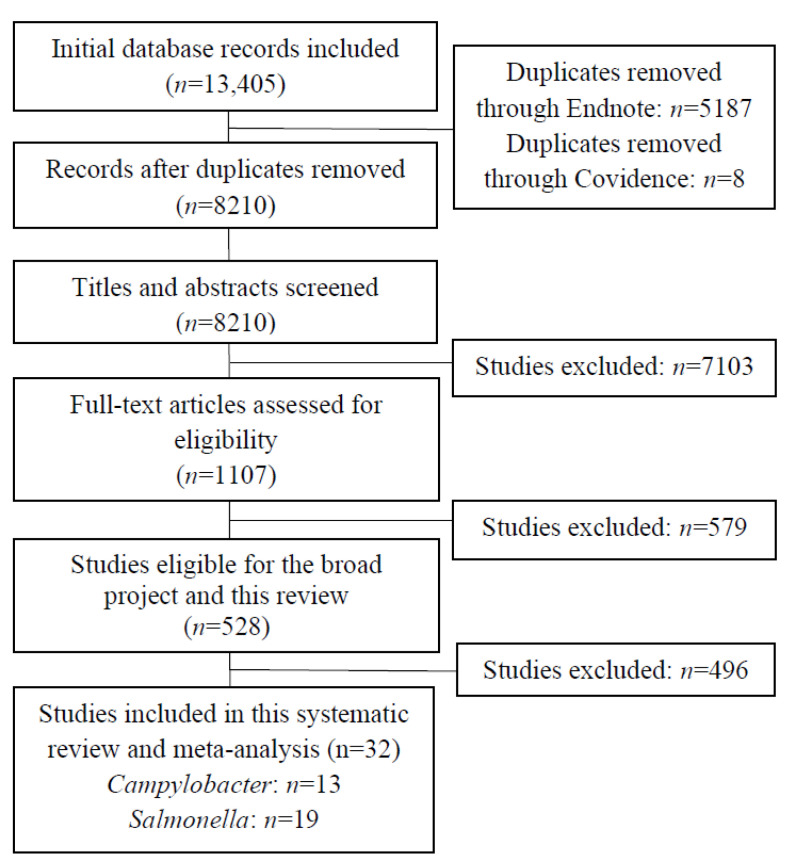
PRISMA flow diagram illustrating the number of studies included at each screening stage.

The search and screening results are summarised in the Preferred Reporting Items for Systematic Reviews and Meta-Analyses (PRISMA) [34] flow diagram (Figure 1). Title and abstract screening were performed on 8210 studies, of which, 404 were from the 2019 to 2022 updated screening (Figure 1). A total of 1107 full texts were screened, including 46 from the updated screening. Of these studies, 579 were excluded due to not satisfying the eligibility criteria. Reasons for exclusion of the full-text studies are summarised in Table 1, with ineligible population (*n* = 143) being the most common reason for exclusion, followed by inaccessible full texts (*n* = 121). A further 496 full-texts were excluded due to not satisfying the eligibility criteria for this review, resulting in a final 32 included in this review, 14 of which were studies from the updated screening.

### 3.2. Characteristics of the Included Studies

The effect of vaccinating meat chickens against *Campylobacter* and *Salmonella* was analysed in 13 and 19 studies, respectively. The characteristics of these studies are summarised in Table 2 and Table 3 for *Campylobacter* and *Salmonella*. A majority of the studies investigating *Campylobacter* vaccines were Canadian (*n* = 4), whereas the majority for *Salmonella* were from the USA (*n* = 8). The remaining studies were from Australia, Belgium, Brazil, Egypt, France, India, Iran, Netherlands and Poland.

All studies used a challenged model experimental design. Studies that investigated *Campylobacter* vaccines used birds challenged with *C. jejuni*. In contrast, different *Salmonella* serovars (Enteritidis, *n* = 9, Typhimurium, *n* = 3, Infantis, *n* = 2, Heidelberg, *n* = 2, or more than one serovar, *n* = 3) were used in *Salmonella* vaccine trials.

Of the 13 *Campylobacter* studies, 7 had a multi-arm treatment experimental design, resulting in a total of 25 outcomes for analysis. Eleven of the nineteen *Salmonella* studies had a multi-arm treatment experimental design, leading to a total of 34 outcomes for analysis. Units of analysis across the studies were either birds or pens. All studies had a cluster-randomised experimental design, approximately half of which had a unit of analysis error.

The most common intervention used across the *Campylobacter* outcomes were subunit vaccines (*n* = 21), followed by inactivated vaccines (*n* = 3) and passive immunisation (*n* = 1). Whereas for *Salmonella*, 17 outcomes used live vaccines, with the remaining interventions being subunit vaccines (*n* = 8), passive immunisation (*n* = 7), an inactivated vaccine (*n* = 1) and a combination of a subunit and live vaccine (*n* = 1).

Bird age at the time of intervention varied across both *Campylobacter* and *Salmonella* outcomes, ranging from being treated in ovo to 3 weeks old. The most common age of intervention for *Campylobacter* outcomes was 6 or 7 days (*n* = 15), whereas for *Salmonella* outcomes, most were first treated at day of hatch or 1 day old (*n* = 25). Birds at the time of outcome assessment were older in the *Campylobacter* outcomes (starting during their third week of age) compared to *Salmonella* outcomes (starting during their first week of age) with 6 and 15 respective outcomes measured at multiple time points.

**Table 2:** Summary of key characteristics of studies that investigated the effects of *Campylobacter* vaccines on caecal *Campylobacter*.

Reference	Country	Population	Intervention	Vaccine Type	Age at Assessment	Challenge	RoB ^1^	Overall RoB
Annamalai et al., 2013 [35]	USA	7 days old Sex and breed not specified	*C. jejuni* outer membrane protein with nanoparticle encapsulation administered via oral gavage	Subunit vaccine	42 days	*C. jejuni*	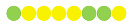	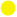 Unclear
Gorain et al., 2020 [36]	India	7 days old Sex not specified Vencobb	(i) Lactococcus lactis expressing functionally active *Campylobacter* JlpA protein administered via oral gavage or (ii) *Campylobacter* JLpA protein emulsified in incomplete Freund’s adjuvant administered via subcutaneous injection	(i and ii) Subunit vaccine	35 days	*C. jejuni*	(i and ii) 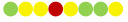	(i and ii) 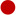 High
Hodgins et al., 2015 [37]	Canada	7 days old Female Ross	Capsular polysaccharide from *C. jejuni* conjugate (i) alone, or with (ii) Addavax™ adjuvant or (iii) CpG adjuvant administered via subcutaneous injection	(i to iii) Subunit vaccine	38 days	*C. jejuni*	(i to iii) 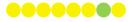	(i to iii) 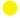 Unclear
Laniewski et al., 2014 [38] ^2^	Poland	1 day old As hatched Cobb	*C. jejuni* cjaA protein carried by S. Typhimurium administered via oral gavage	Subunit vaccine	35 and 42 days	*C. jejuni*	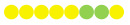	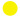 Unclear
Meunier et al., 2018 [39]	France	1 day old Mixed sex Ross	*C. jejuni (i)* pcDNA3-*flaA* or (ii and iii) recFlaA carried by *E. coli* administered via (i) subcutaneous or (ii and iii) intramuscular injection	(i to iii) Subunit vaccine	28 and 42 days	*C. jejuni*	(i to iii) 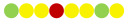	(i to iii) 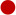 High
Neal-McKinney et al., 2014 [40]	USA	6 days old Sex not specified Cornish Cross	GST-tagged 90 mer peptides boosted with *C. jejuni* surface-exposed colonisation proteins (i) CadF, (ii) FlaA, (iii) FlpA, (iv) Trifecta or (v) CmeC administered via subcutaneous injection	(i to v) Subunit vaccine	27 days	*C. jejuni*	(i to v) 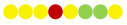	(i to v) 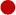 High
Nothaft et al., 2017 [41]	Canada	7 days old Sex not specified Ross 308	*E. coli* expressing *C. jejuni* protein administered via oral gavage	Subunit vaccine	35 days	*C. jejuni*	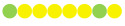	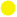 Unclear
Nothaft et al., 2021 [42] ^2^	Canada	7 days old Sex not specified Ross 308	*E. coli* expressing the *C. jejuni* N-glycan administered via oral gavage	Subunit vaccine	35 days	*C. jejuni*	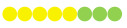	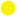 Unclear
Radomska et al., 2016 [43] ^2^	Netherlands	18d egg incubation As hatched Ross 308	*C. jejuni* flagellin-based vaccine administered in ovo	Subunit vaccine	25 days	*C. jejuni*	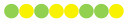	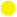 Unclear
Singh et al., 2019 [44]	India	7 days old Sex not specified Vencobb	*E. coli* expressing *C. jejuni* hcp protein (i) entrapped in chitosan cross-linked with sodium tripolyphosphate nanoparticles administered via oral gavage or (ii) emulsified with IFA and administered via subcutaneous injection	(i and ii) Subunit vaccine	28 days	*C. jejuni*	(i) 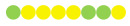 (ii) 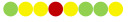	(i) 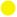 Unclear (ii) 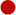 High
Taha-Abdelaziz et al., 2018 [45]	Canada	14 days old Sex not specified Ross 708	*C. jejuni* lysate protein (i) without or (ii) with PLGA-encapsulated CpG administered via oral gavage	(i and ii) Inactivated vaccine	23, 30 and 37 days	*C. jejuni*	(i and ii) 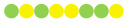	(i and ii) 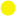 Unclear
Vandeputte et al., 2019 [46] ^2^	Belgium	15d egg incubation As hatched Ross 308	(i) *C. jejuni* bacterin vaccine or (ii) a subunit vaccine containing 6 *Campylobacter* antigens administered in ovo	(i) Inactivated vaccine(ii) Subunit vaccine	24 days	*C. jejuni*	(i and ii) 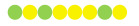	(i and ii) 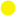 Unclear
Vandeputte et al., 2020 [47] ^2^	Belgium	Day of hatch As hatched Ross 308	Hyperimmune egg yolk powder against *C. jejuni* and *C. coli* strains administered in feed	Passive immunisation	16 days	*C. jejuni*	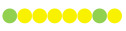	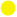 Unclear

^1^ Risk of bias was assessed in the order of: Sequence generation, Allocation concealment, Timing of identification and Recruitment of individual participants, Blinding of participants and personnel, Blinding of outcome assessors, Incomplete outcome data, Selective outcome reporting and Other sources of bias. Assessment for each domain is represented in green 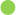 (low risk of bias), yellow 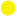 (unclear risk of bias) and red 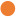 (high risk of bias). ^2^ Studies that did not have a unit of analysis error. Note: Population age refers to the bird’s age at the beginning of the intervention.

**Table 3:** Summary of key characteristics of studies that investigated the effects of *Salmonella* vaccines on caecal *Salmonella*.

Reference	Country	Population	Intervention	Vaccine Type	Age at Assessment	Challenge	RoB ^1^	Overall RoB
Acevedo-Villanueva et al., 2020 [48] ^2^	USA	1 day old Sex not specified Cobb-500	(i) *S.* Enteritidis chitosan-nanoparticle vaccine or (ii) Poulvac^®^ ST administered orally and challenged with *S.* Heidelberg or *S.* Enteritidis	(i) Subunit vaccine (ii) Live vaccine	16 days	*Salmonella* Enteritidisor Heidelberg	(i and ii) 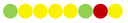	(i and ii) 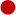 High
Acevedo-Villanueva et al., 2021a [49] ^2^	USA	18d egg incubation Sex N/A Cobb	*S.* Enteritidis chitosan-nanoparticle vaccine administered in ovo	Subunit vaccine	14 days and 21 days	*Salmonella* Enteritidis	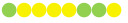	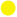 Unclear
Acevedo-Villanueva et al., 2021b [50] ^2^	USA	1 day old Sex not specified Cobb	(i) *S.* Enteritidis chitosan-nanoparticle (CNP) vaccine, or (ii) Poulvac^®^ ST, or (iii) Poulvac^®^ ST boosted with CNP vaccine, all administered orally	(i) Subunit vaccine (ii) Live vaccine (iii) Subunit and live vaccine	28 days	*Salmonella* Enteritidis	(i to iii) 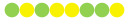	(i to iii) 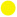 Unclear
Chalghoumi et al., 2009 [51] ^2^	Belgium	1 day old Male Ross	Hyperimmune egg yolk powder administered in feed	Passive immunisation	7, 14, 21 and 28 days	*Salmonella* Enteritidis and Typhimurium	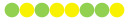	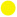 Unclear
De Cort et al., 2013 [52]	Belgium	1 day old Sex not specified Ross	*S.* Enteritidis 76Sa88 hilAssrAfliG deletion mutant administered via oral gavage	Live vaccine	7, 21 and 42 days	*Salmonella* Enteritidis	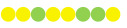	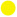 Unclear
De Cort et al., 2014 [53]	Belgium	1 day old Sex not specified Ross	(i) hilAssrAfliG deletion mutant (*S.* Typhimurium) or (ii) hilAssrAfliG deletion mutant (*S.* Typhimurium and *S.* Enteritidis) administered via oral gavage	(i and ii) Live vaccine	(i) 7, 21 and 42 days old or (ii) 7 days	*Salmonella* Typhimurium, Enteritidis and Paratyphi Java	(i and ii) 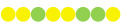	(i and ii) 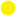 Unclear
De Cort et al., 2015 [54]	Belgium	1 day old Sex not specified Ross 308	*S.* Enteritidis Delta hilAssrAfliG strain administered in (i) drinking water or (ii) sprayed	(i and ii) Live vaccine	7, 21 and 42 days	*Salmonella* Enteritidis	(i) 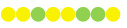 (ii) 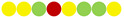	(i) 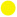 Unclear (ii) 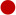 High
El-Ghany et al., 2012 [55]	Egypt	1 day old Mixed sex Hubbard	*S.* Enteritidis bacterin administered via intramuscular injection	Inactivated vaccine	27, 34 and 41 days	*S.* Enteritidis	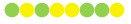	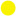 Unclear
El-Shall et al., 2020 [56]	Egypt	7 days old Sex not specified Cobb	*S.* Enteritidis vaccine administered via drinking water (i) with and (ii) without *S.* Enteritidis challenge	(i and ii) Live vaccine	35 days and 42 days	*Salmonella* Enteritidis	(i and ii) 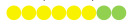	(i and ii) 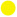 Unclear
Han et al., 2020a [57]	USA	3 days old or 3 weeks old Sex not specified Cornish Criss	*S.* Enteritidis chitosan-nanoparticle vaccine administered at (i) 3 days or (ii) 3 weeks of age via oral gavage	(i and ii) Subunit vaccine	45 days	*Salmonella* Enteritidis	(i and ii) 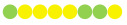	(i and ii) 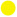 Unclear
Han et al., 2020b [58]	USA	3 days old Sex not specified Cornish Cross	*S.* Enteritidis chitosan-nanoparticle vaccine (i) with flagellin surface-coating or (ii) mannose-ligand-modification instead of flagellin surface-coating or (iii) with both flagellin surface-coating and mannose-ligand modification or (iv) Poulvac^®^ ST administered orally	(i to iii) Subunit vaccine (iv) Live vaccine	45 days	*Salmonella* Enteritidis	(i to iv) 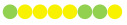	(i to iv) 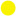 Unclear
Isfahani et al., 2020 [59] ^2^	Iran	1 day old Male Cobb-500	(i) *Salmonella* immune powdered yolk or (ii) *Salmonella* capsulated immune yolk administered in feed	(i and ii) Passive immunisation	7, 14 and 21 days	*Salmonella* Infantis	(i and ii) 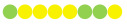	(i and ii) 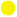 Unclear
Jones et al., 2021 [60]	USA	Day of hatch Straight run (i and iii) Ross 708 (ii) Cobb 500	(i and ii) Poulvac^®^ ST boosted by gavage, or (iii) Poulvac^®^ ST boosted via drinking water	(i to iii) Live vaccine	(i) 46 days (ii) 43 days (iii) 40 and 41 days	*Salmonella* Infantis	(i to iii) 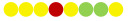	(i to iii) 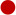 High
Muniz et al., 2017 [61] ^2^	Brazil	1 day old Mixed sex Cobb slow growing	Poulvac^®^—ST sprayed	Live vaccine	28 days	*Salmonella* Heidelberg	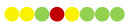	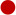 High
Pavic et al., 2010 [62]	Australia	1 day old AH Cobb	Parent bird injected with trivalent inactivated vaccine	Passive immunisation	21 days	*Salmonella* Typhimurium	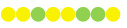	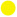 Unclear
Rubinelli et al., 2015 [63]	USA	Day of hatch Male Cobb	Pbad-mviN *Salmonella* vaccine administered via oral gavage	Live vaccine	42 days	*Salmonella* Typhimurium	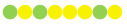	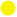 Unclear
Teixeira et al., 2022 [64]	Brazil	1 day old As hatched Cobb-500	Birds were progeny from breeders vaccinated with (i) Gallimune^®^ SE + ST or (ii) Nobilis^®^ Salenvac T	(i and ii) Passive immunisation	4, 6, 9, 15 and 23 days	*Salmonella* Heidelberg	(i and ii) 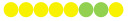	(i and ii) 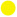 Unclear
Wolfenden et al., 2010 [65]	USA	Day of hatch Sex not specified Cobb	*S.* Enteritidis vector strain (ΔSEEV) with modified variants expressing (i) a flagellar filament protein (fliC) antigen alone (SEΔFliC) or (ii) with a putative immunopotentiating compound CD154 (SEΔfliC-CD154C) administered via oral gavage	(i and ii) Live vaccine	10, 19 and 30 days	*Salmonella* Typhimurium	(i and ii) 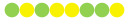	(i and ii) 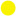 Unclear
Yamawaki et al., 2021 [66]	Brazil	1 day old As hatched Breed not specified	Birds were progeny from breeders vaccinated with recombinant vaccine	Passive immunisation	3 and 6 days	*Salmonella* Enteritidis	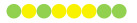	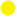 Unclear

^1^ Risk of bias was assessed in the order of: Sequence generation, Allocation concealment, Timing of identification and Recruitment of individual participants, Blinding of participants and personnel, Blinding of outcome assessors, Incomplete outcome data, Selective outcome reporting and Other sources of bias. Assessment for each domain is represented in green 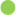 (low risk of bias), yellow 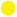 (unclear risk of bias) and red 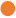 (high risk of bias). ^2^ Studies that did not have a unit of analysis error. Note: Population age refers to the bird’s age at the beginning of the intervention.

### 3.3. Risk of Bias

The risk of bias assessed for the eight domains was used to give an overall judgment for each outcome resulting in most being unclear (Table 2 and Table 3). A noticeable lack of detailed methods and results was the main contributor for the unclear assessment across various domains, specifically, allocation concealment, blinding of personnel administering interventions and blinding of personnel assessing outcomes. No studies addressed any method of blinding or concealment in their methods. No outcomes were judged to have an overall low risk of bias.

*Campylobacter* had 10 outcomes judged with an overall high risk of bias (Table 2). This was due to visibly different methods of administrating treatments in a multi-arm study, such as administering one treatment via oral gavage vs. subcutaneous injection [36,39]. Another cause was not giving the control group a sham treatment to replicate a visible method of administering treatment, such as administering treatments via subcutaneous injection and not injecting birds with a diluent (e.g., phosphate-buffered saline) [40,44].

Similar reasons for high risk of bias were found in five *Salmonella* outcomes (Table 3) where control groups were not treated with a sham treatment method [61] or multi-arm studies administered treatments via different visible methods [54,60]. An additional study, however, was judged to have a high risk of bias for two outcomes due to selective outcome reporting [48]. Although the study measured the effects of both treatments on caecal *Salmonella* at days 16 and 18, the outcomes were only reported at day 16.

Overall, selective outcome reporting was the most common domain judged as having a low risk of bias across both *Campylobacter* and *Salmonella* outcomes, followed by incomplete outcome data. This indicated a common absence of detailed intervention methods.

### 3.4. Intervention Effects

**Figure 2:**
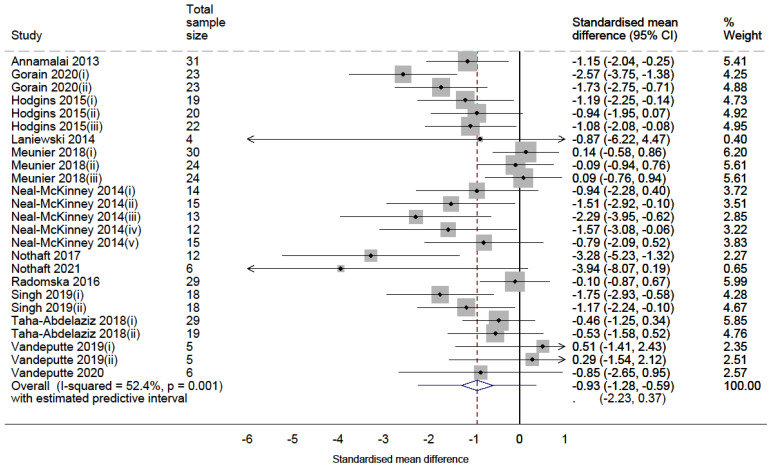
Random effects meta-analysis forest plot synthesising the effects of vaccinating meat chickens against *Campylobacter* [35,36,37,38,39,40,41,42,43,44,45,46,47]. Each point represents the calculated SMD of each comparator with whiskers representing the 95% confidence interval. Each box represents the weight percentage that each study contributes to the final pooled estimate. The middle of the diamond (bottom) represents the pooled estimate, and the sides represent its 95% confidence interval. The whiskers at either side of the diamond represent the estimated predictive interval.

**Figure 3:**
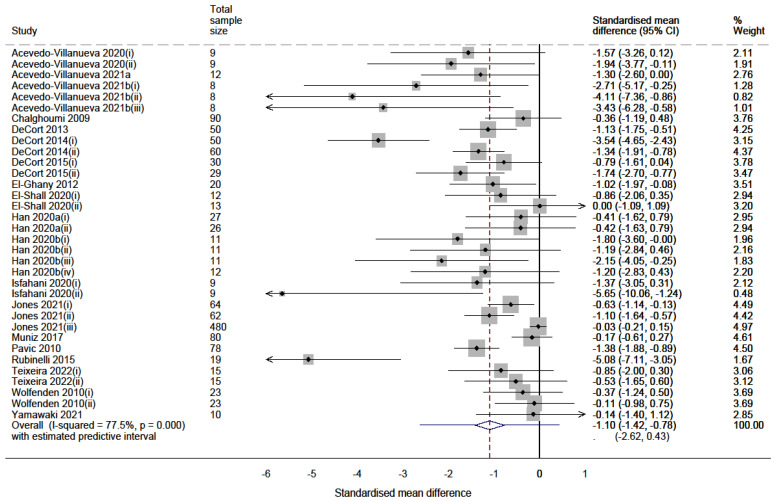
Random effects meta-analysis forest plot synthesising the effects of vaccinating meat chickens against *Salmonella* [48,49,50,51,52,53,54,55,56,57,58,59,60,61,62,63,64,65,66]. Each point represents the calculated SMD of each comparator with whiskers representing the 95% confidence interval. Each box represents the weight percentage that each study contributes to the final pooled estimate. The middle of the diamond (bottom) represents the pooled estimate, and the sides represent its 95% confidence interval. The whiskers at either side of the diamond represent the estimated predictive interval.

The random effects meta-analyses for both vaccine syntheses revealed an overall reduction of *Campylobacter* and *Salmonella* in the caeca of meat chickens. Forest plots of the *Campylobacter* and *Salmonella* meta-analyses are shown in Figure 2 and Figure 3, respectively, and summarised in Table 4.

The meta-analysis of comparators investigating the use of vaccines on *Campylobacter* (Figure 2) included 25 comparators and estimated a significant average reduction of 0.93 standard deviation units (SMD = −0.93; 95% CI = −1.275 to −0.585; *p* value < 0.001). The meta-analysis investigating the use of vaccines on *Salmonella* (Figure 3) (*n* = 34 comparators) estimated a similarly significant average reduction of 1.10 standard deviation units (SMD = −1.10; 95% CI = −1.419 to −0.776; *p* value < 0.001).

**Table 4:** Statistical results for random effects meta-analyses of standardised mean differences to investigate the effect of vaccinations on caecal *Campylobacter* and *Salmonella* in meat chickens. SMDs are presented to 2 decimal places.

	Meta-Analysis	N	SMD	95% Confidence Interval	τ^2^ *	Z-Test *p* Value ^†^	I^2^ (%) ^‡^	Chi^2^ *p* Value ^§^
***Campylobacter***								
Primary random effects analysis		25	−0.93	−1.275 to −0.585	0.365	<0.001	52.4	0.001
Sensitivity random effects analysis (ICC = 0.1)		25	−0.86	−1.187 to −0.523	0.171	<0.001	25.3	0.123
Group	Subunit vaccine	21	−1.04	−1.444 to −0.645	0.452	<0.001	57.8	0.001
	Inactivated vaccine	3	−0.39	−0.987 to 0.218	<0.001	0.211	<0.1	0.624
	Passive immunisation	1	−0.85	−2.652 to 0.947	<0.001	0.353	N/A	N/A
***Salmonella***								
Primary random effects analysis		34	−1.10	−1.419 to −0.776	0.533	<0.001	77.5	<0.001
Sensitivity random effects analysis (ICC = 0.1)		34	−0.86	−1.176 to −0.539	0.254	<0.001	43.6	<0.001
Group	Subunit vaccine	8	−1.15	−1.685 to −0.605	<0.001	<0.001	<0.1	0.512
	Inactivated vaccine	1	−1.02	−1.970 to −0.078	<0.001	0.034	N/A	N/A
	Passive immunisation	7	−0.88	−1.462 to −0.300	0.260	0.003	48.1	0.073
	Live vaccine	17	−1.10	−1.541 to −0.648	0.614	<0.001	85.5	<0.001
	Subunit and Live vaccine	1	−3.43	−6.283 to −0.580	<0.001	0.018	N/A	N/A

* τ^2^ represents the estimated between-study variance. Statistics are presented to 3 decimal places. ^†^ Z-test is a significance test for the weighted average effect size. Statistics are presented to 3 decimal places. ^‡^ I^2^ percentage represents the level of variation in SMDs attributable to heterogeneity. Statistics are presented to 1 decimal place. ^§^ Chi^2^ test is a significance test for the heterogeneity between studies. Statistics are presented to 3 decimal places.

#### 3.4.1. Heterogeneity

Although the confidence intervals of the pooled effects were negative for both syntheses, significant levels of heterogeneity were present (Chi^2^ *p* values = 0.001 and <0.001 for *Campylobacter* and *Salmonella*, respectively). The I^2^ statistic was 52.4% for the *Campylobacter* synthesis and 77.5% for the *Salmonella* synthesis, interpreted as substantial heterogeneity of effects for both syntheses. The variability of effects contributed to wide prediction intervals of effects in similar studies, some of which may increase *Campylobacter* (−2.23 to 0.37) or *Salmonella* (−2.62 to 0.43).

#### 3.4.2. Reporting Bias

**Figure 4:**
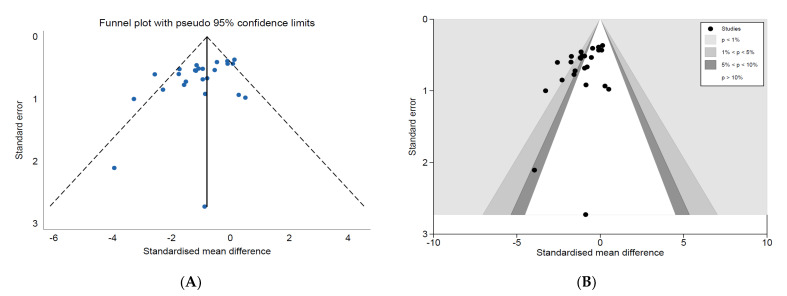
(**A**) Standard and (**B**) contour-enhanced funnel plots for comparators investigating the effects of vaccinating meat chickens against *Campylobacter*. Note: (**A**) Lower and upper 95% confidence intervals are represented by the dashed lines and the pooled SMD represented by the vertical solid line; (**B**) white/no shading: *p* > 10%, dark grey: 5% < *p* < 10%, grey: 1% < *p* < 5%, light grey: *p* < 1%.

**Figure 5:**
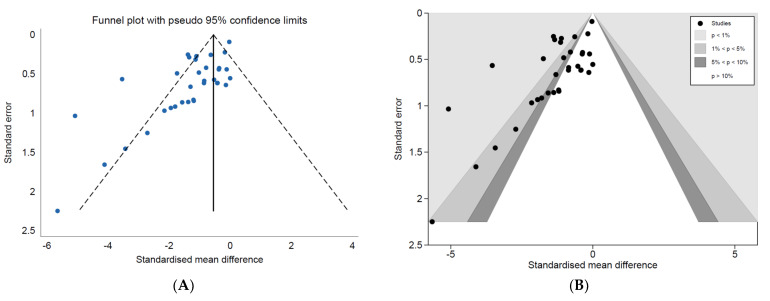
(**A**) Standard and (**B**) contour-enhanced funnel plots for comparators investigating the effects of vaccinating meat chickens against *Salmonella.* Note: (**A**) Lower and upper 95% confidence intervals are represented by the dashed lines and the pooled SMD represented by the vertical solid line; (**B**) white/no shading: *p* > 10%, dark grey: 5% < *p* < 10%, grey: 1% < *p* < 5%, light grey: *p* < 1%.

The standardised effects were plotted against their standard errors in inversed funnel plots to identify the presence of reporting bias (Figure 4 and Figure 5). Funnel plots for both analyses were asymmetrical, leaning towards the upper left region, most obvious in the *Salmonella* analysis (Figure 5). The contour-enhanced funnel plot for the *Campylobacter* analysis (Figure 4B) showed studies to have an approximately equal distribution in all levels of statistical significance. However, the gap in the middle to right region of the funnel plot for *Salmonella* and the missing smaller studies in regions of statistical non-significance (Figure 5) indicated the presence of publication bias.

#### 3.4.3. Subgroup Analysis

Random effects subgroup meta-analyses and meta-regression were performed to investigate whether the different intervention types were a contributing factor causing the observed statistical heterogeneity. Comparators across both the *Campylobacter* and *Salmonella* analyses were grouped by the vaccination or immunisation type, and results are summarised in Table 4.

The SMDs used for the *Campylobacter* synthesis were categorised into three intervention types: subunit vaccine (*n* = 21), inactivated vaccine (*n* = 3) and passive immunisation (*n* = 1). The inconsistency of comparators, represented by the I^2^ statistic, was substantial for subunit vaccines (I^2^ = 57.8%) and minor for inactivated vaccines (I^2^ < 0.1%).

The SMDs used for the *Salmonella* synthesis were categorised into five intervention types: subunit vaccine (*n* = 8), inactivated vaccine (*n* = 1), passive immunisation (*n* = 7), live vaccine (*n* = 17) and subunit with live vaccine (*n* = 1). A considerably high level of heterogeneity of effects was observed within the live vaccine group (I^2^ = 85.5%). The inconsistency was moderate for the effects of passive immunisation (I^2^ = 48.1%) and minor for the effects of subunit vaccines (I^2^ < 0.1%).

The difference in SMDs across the subgroups was estimated in a random effects meta-regression (Table 5). A large amount of variability remaining in the meta-regression model for *Campylobacter* vaccines (Residual I^2^ = 54.5%) indicated that the type of interventions administered was not the major source of heterogeneity (Bonferroni adjusted *p* value = 1; Unadjusted *p* value = 0.410). The difference in effects was variable when compared to the reference subunit vaccine group; however, the number of comparators within the inactivated vaccine and passive immunisation group was much lower than the subunit group.

A high level of variability remained in the meta-regression model for *Salmonella* vaccines (Residual I^2^ = 77.4%) with little evidence suggesting the type of interventions could explain the heterogeneity in the synthesised effect (Bonferroni adjusted *p* value = 1; Unadjusted *p* value = 0.733). Absolute deviations from the reference subunit vaccine group were less than 0.40 SMD units, except for the combined subunit and live vaccine group containing one comparator with a very wide confidence interval.

**Table 5:** Summarised results of the meta-regression for vaccinations against *Campylobacter* (*n* = 25) and *Salmonella* (*n* = 34) comparators subgrouped by vaccine or immunisation type. SMDs and *p* values are presented to 2 decimal places, and 95% CI’s are presented to 3 decimal places.

Intervention Type	Difference in SMDs	95% Confidence Interval	*p* value
***Campylobacter***			
Subunit vaccine (Reference intervention type)	-	-	-
Inactivated vaccine	0.73	−0.382 to 1.838	0.19
Passive immunisation	0.18	−2.158 to 2.519	0.87
***Salmonella***			
Subunit vaccine (Reference intervention type)	-	-	-
Inactivated vaccine	0.25	−1.994 to 2.499	0.82
Passive immunisation	0.37	−0.872 to 1.622	0.54
Live vaccine	0.19	−0.846 to 1.222	0.71
Subunit and live vaccine	−2.16	−6.000 to 1.690	0.26

#### 3.4.4. Sensitivity Analysis

To examine the effects of within-cluster correlations for studies that had a unit of analysis error, an ICC of 0.1 was used to estimate effective sample sizes. New statistically adjusted SMDs were estimated for 19 *Campylobacter* comparators and 24 *Salmonella* comparators.

A random effects meta-analysis synthesising the adjusted *Campylobacter* SMDs estimated a pooled SMD of −0.86 (95% CI: −1.187 to −0.523; *p* value < 0.001). Compared to the primary meta-analysis (Table 4), the difference in effects was small (absolute SMD difference = 0.07), with a narrower confidence interval and lower levels of heterogeneity (I^2^ = 25.3%; Chi^2^ *p* value = 0.123).

The adjusted (ICC = 0.1) random effects meta-analysis synthesising the effects of *Salmonella* vaccines estimated an average *Salmonella* reduction of 0.86 SMD units (SMD = −0.86; 95% CI: −1.176 to −0.539; *p* value < 0.001). The absolute difference in effects (0.24 SMD units) was slightly larger than that observed for the *Campylobacter* synthesis. Although a significant level of heterogeneity remained (I^2^ = 43.6; Chi^2^ *p* value < 0.001), the analysis still indicated that the use of vaccines resulted in a significantly large average reduction of *Salmonella*.

The impact of unit of analysis errors in the syntheses was small. The magnitude of effects was reduced in both adjusted syntheses; however, a high reduction of *Campylobacter* and *Salmonella* remained.

### 3.5. Confidence in the Body of Evidence

The level of confidence in the body of evidence for both syntheses was evaluated using the GRADE approach. The consistent unclear assessment in the outcome risk of bias due to intervention methods led to a high overall risk of bias for both syntheses. This high risk of bias in the pooled effect reduced the confidence in evidence by one level. There was a high level of inconsistency in the syntheses that could not be explained through subgroup analysis. As the heterogeneity of effects could not be explained, the confidence in the findings of both syntheses was reduced by one level. The higher level of inconsistency in the *Salmonella* synthesis led to a further reduction to a very low level of confidence. The indirectness of the included studies was low as studies fit within the pre-specified range of eligible populations, interventions, comparators and outcomes. The level of imprecision of the pooled effects was also low as the 95% confidence intervals excluded the null (SMD = 0) effect of vaccinations and both upper and lower limits were negative. The confidence in the body of evidence was not reduced for indirectness or imprecision in both syntheses. Publication bias, revealed by a gap of smaller studies in areas of statistical non-significance, was present in the *Salmonella* synthesis. This selective suppression led to a further reduction of confidence by one level. The overall certainty of gathered evidence was increased by one level for the syntheses due to the large magnitude of effects. This resulted in a moderate level of confidence in the *Campylobacter* vaccine effect estimate, meaning the true effect is likely close to the estimated effect. For *Salmonella*, however, there was a very low level of confidence in the effect estimate, meaning the true effect is likely smaller than what was estimated.

## 4. Discussion

A systematic review and meta-analyses were performed to determine the effects of vaccines on caecal *Campylobacter* and *Salmonella* in meat chickens. The meta-analyses indicated that vaccines were effective in reducing *Campylobacter* and *Salmonella* in the caeca of meat chickens. However, the confidence in findings was limited by high levels of heterogeneity and consistent risk of bias in the study design. Publication bias was also found in the *Salmonella* review, further reducing the confidence in findings. This resulted in a moderate level of confidence in the estimated effects on *Campylobacter* and a very low level of confidence in the estimated effects on *Salmonella*.

Adjustment for unit of analysis errors was performed using an ICC of 0.1 to calculate effective sample sizes, and the synthesised adjusted effects were not substantially different to the unadjusted estimates. Due to the reduction of sample sizes, standard errors of the SMDs were inflated, contributing to less heterogeneity in the synthesis. It is possible that the correlation that existed within the unadjusted clustered groups may not only have exaggerated the effect of the treatments but may also have contributed to the heterogeneity between the different studies. As the ICC is likely to vary with study characteristics such as challenge status, ages of birds and interventions used [67], results should be interpreted cautiously as the ICC used may not have been appropriate for all the studies included. Although further research into estimating a variety of appropriate ICCs to analyse cluster correlated data would be useful, the prevention of unit of analysis errors at the experimental design stage is recommended for future research.

Many studies (*n* = 121) were excluded from the broad review due to unavailable full texts. It is unclear how many of these studies would have satisfied the eligibility criteria for this review. A larger number of studies would also have facilitated an improved subgroup analysis investigation. It is also worth noting the variety of *Salmonella* serovars investigated in the literature compared to the one *Campylobacter* species. Although approximately 90% of campylobacteriosis is caused by *C. jejuni*, other species such as *C*. *coli* and *C. lari* can also cause human illness [68].

Most of the *Campylobacter* vaccine types were subunit vaccines, whereas majority of those for *Salmonella* were live vaccines. The use of live vaccines is generally considered to be the most effective immunisation type for pathogen protection; however, it also introduces the risk of colonising the host [69]. This is especially true for *Campylobacter*, as chickens can very easily become colonised with high numbers of *C. jejuni* in the intestinal tract [37]. There is limited literature on comparing the efficacy of different *Campylobacter* and *Salmonella* vaccine types; however, evidence supports a strong but short-lived response to inactivated vaccines [70]. Key disadvantages of inactivated *Salmonella* vaccines are that they require the use of adjuvants, are less likely to carry their beneficial effects to progeny and carry a risk of improper inactivation [70,71]. Although subunit vaccines are comparatively newer in development and require the use of an adjuvant, subunit vaccines are composed of defined antigens making them the safest choice of the three [71].

Although the subgroup analyses did not unveil any obvious differences between the vaccine types, there were less than the recommended 10 comparators in the remaining subgroups to conclude robust findings for both syntheses [72,73]. Amongst the vaccine types, the effects of *Salmonella* subunit vaccines had a comparatively low level of heterogeneity with a promising beneficial effect on caecal *Salmonella*. While no significant differences between the subgroups were seen in the meta-regression for both *Campylobacter* and *Salmonella*, conclusions should be interpreted with caution due to the observational nature of the meta-regression as well as the low number of comparators within subgroups [72,73]. There are many factors within each study setting that could have contributed to the different effects. As the intervention types were the most anticipated source of heterogeneity, no other subgroup analysis was conducted to prevent data dredging.

It is possible that many of the unclear risk of bias assessments could have been resolved if the authors were contacted for clarification, however, this review intended to evaluate the findings in the readily available literature. The high risk of bias due to the use of visibly different intervention methods can sometimes be avoided using sham methods, however, in other cases, unavoidable especially when the application method of the treatment is what is being investigated. Fulfilling the reporting guidelines recommended in the REFLECT statement (Reporting guidelines for randomized control trials in livestock and food safety) [74] is not common practice in animal experiments, however, would be incredibly beneficial to improve study risk of bias. Registering the trials in an animal registry potentially could have corrected the issue of publication bias apparent in the *Salmonella* synthesis [75]. Although this is also not common practice, the use of trial registers can also improve transparency in reporting methods, animal welfare and study reproducibility [76].

Consideration of experimental units of analysis, fulfilment of recommended reporting guidelines and addressing publication bias is necessary to improve the certainty of evidence for the evaluation of vaccinations against *Campylobacter* and *Salmonella* in meat chickens.

## 5. Conclusions

Meta-analysing the effects of vaccines against caecal *Campylobacter* and *Salmonella* revealed a large reduction in pathogen levels, however, with different levels of confidence in the findings. There was a moderate level of confidence in the reduction of *Campylobacter* levels by 0.93 SMD units (95% CI: −1.275 to −0.585; *p* value < 0.001) and a very low level of confidence in the reduction of *Salmonella* by 1.10 SMD units (95% CI: −1.419 to −0.776; *p* value < 0.001). Higher levels of heterogeneity and publication bias were the main limiting factors for the confidence in *Salmonella* findings. High levels of heterogeneity in both syntheses contributed to wide prediction intervals encompassing a majority of overall beneficial effects, however, were not able to predict similar individual studies will always reduce *Campylobacter* or *Salmonella*. Further research into vaccinations for *Campylobacter* and *Salmonella* is essential to source areas of heterogeneity. It is likely further research will also support the beneficial and consistent effects of *Salmonella* subunit vaccines seen in this review. Consideration of experimental units of analysis, a commitment to reporting guidelines and registration of trials is necessary to improve the levels of confidence in future research.

## Figures and Tables

**Table 1:** Number of studies excluded at the full-text screening stage with justification. Note: one reason of exclusion was assigned to each excluded study. Values include studies from the updated vaccine search excluded due to article type (*n* = 1), irrelevant population (*n* = 18), wrong intervention (*n* = 3), irrelevant outcome (*n* = 9) and unclear intervention (*n* = 1).

Number of Studies Excluded	Justification for Exclusion of Study
6	Duplicate Study
31	Not in English
6	Article Type: Not Primary Research
121	Full Text Unobtainable
143	[POPULATION] Population Irrelevant
56	[POPULATION] Setting Irrelevant (Not Production/Processing)
32	[INTERVENTION] No or Wrong Intervention
33	[COMPARATOR] No Concurrent Comparator or Control
61	[OUTCOME] Outcome Irrelevant (Not *Campylobacter* and *Salmonella*)
38	[OUTCOME] Reported outcomes insufficiently detailed
39	[METHODS] Insufficient Detail for Replication of Study
13	[METHODS] Laboratory Methods Inappropriate
579	Total number of studies excluded during full-text screening

## Data Availability

Not applicable.
